# Middle Ear Ceruminous Gland Adenoma Obstructing the Eustachian Tube Orifice

**DOI:** 10.1155/2021/5987353

**Published:** 2021-07-14

**Authors:** Hamin Jeong, Haemin Noh, Chang-Hee Kim

**Affiliations:** Department of Otorhinolaryngology-Head and Neck Surgery, Konkuk University Medical Center, Research Institute of Medical Science, Konkuk University School of Medicine, Seoul, Republic of Korea

## Abstract

Ceruminous glands are located in the skin of the cartilaginous portion of the external auditory canal, and ceruminous gland adenoma originating from the middle ear mucosa is extremely rare. We report a case of middle ear ceruminous gland adenoma which caused long-standing otomastoiditis and mixed hearing loss with a large air-bone gap by obstructing the bony Eustachian tube. We discuss the clinical characteristics and histologic features of the present case.

## 1. Introduction

Cerumen, which plays an important role in protecting the ear from infection and mechanical damage, is produced by the ceruminous gland and sebaceous gland. The human ceruminous glands are modified apocrine glands and located in the skin of the cartilaginous portion of the external auditory canal [1, 2]. The ceruminous gland neoplasms in the middle ear cavity are extremely rare because ceruminous glands are not distributed in the middle ear cavity, and only few cases have been reported in the English literature [3–10]. In the present study, we report a case of the middle ear ceruminous gland adenoma which caused long-standing otomastoiditis and conductive hearing loss by obstructing the Eustachian tube orifice. This work has been reported in line with the SCARE guidelines [11].

## 2. Case Presentation

**Figure 1:**
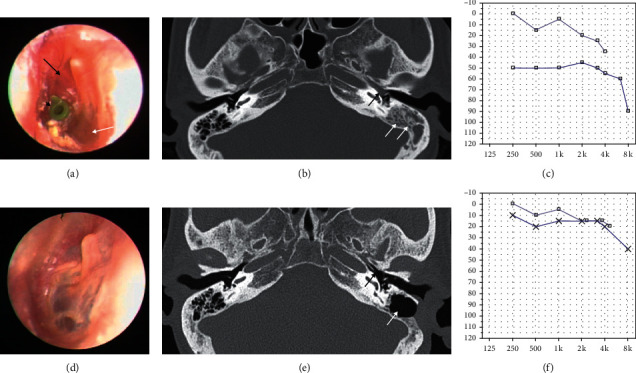
Preoperative (a–c) and postoperative (d–f) clinical findings. (a) Preoperative otoendoscopic examination revealed a pinkish mass-like lesion (black arrow) behind the anterior portion of the tympanic membrane. Middle ear effusion is observed (white arrow) despite previous ventilation tube insertion (black arrow head). (b) Axial view of temporal bone computed tomography (TBCT) demonstrated soft tissue density obstructing the bony portion of the Eustachian tube (black arrow) and mastoiditis (white arrows). (c) Pure tone audiometry showed conductive hearing loss with an air-bone gap of 37 dB in the left side. (d) Postoperative otoendoscopic examination revealed normal tympanic membrane. (e) Axial view of TBCT demonstrated a clean mastoid cavity (white arrow) and no residual mass in the bony portion of the Eustachian tube (black arrow). (f) Pure tone audiometry showed that the air-bone gap was reduced to 5 dB in the left side.

**Figure 2:**
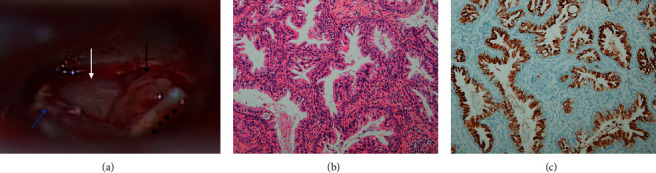
(a) A nonencapsulated, polypoid, pinkish mass (black arrow) and gray-colored mass with fibrotic consistency (white arrow) were seen in the anterior part of the tympanic cavity. Black arrow heads, malleus handle; blue arrow, tympanomeatal flap. (b) Tubular tumor cell structures with intervening bands of tumor stroma are observed, hematoxylin-eosin X100. (c) Immunohistochemical staining for cytokeratin 7 was positive in luminal cells, X100.

A previously healthy 56-year-old woman complained of left-side hearing loss over a 10-month period. She had been receiving treatment at another hospital and undergone ventilation tube insertion surgery in the left ear 4 month prior. The patient reported that the left-side hearing loss was not relieved after the surgery. On the otoendoscopic examination, a pinkish mass was seen through the anterosuperior quadrant of the tympanic membrane without discharge (Figure 1(a)). A nonenhanced temporal bone computed tomography (TBCT) demonstrated soft tissue density obstructing the bony portion of the Eustachian tube and opacification in the middle ear and mastoid cavity with an intact bony labyrinth (Figure 1(b)). A pure tone audiometry (PTA) revealed the left-side mixed hearing loss with a large air-bone gap (Figure 1(c)). A retroauricular canal wall-up tympanomastoidectomy was performed, and a polypoid mass filling the anterior part of the tympanic cavity was revealed (Figure 2(a)) and completely removed. Histological examination revealed a nonencapsulated mass composed of glandular structures lined by two layers of epithelium originating from the ceruminous gland, which was consistent with ceruminous gland adenoma (Figure 2(b)). Luminal cells were positive for cytokeratin 7 (Figure 2(c)). One year after surgery, the left tympanic membrane appeared normal (Figure 1(d)), TBCT revealed no residual mass in the tympanic cavity (Figure 1(e)), and conductive hearing loss was much improved (Figure 1(f)).

## 3. Discussion

Although ceruminous gland neoplasms are relatively common in other mammals [12], they are highly uncommon in humans [2]. Ceruminous glands are primarily located in the skin lining cartilaginous portion of the external auditory canal, and most tumors originating from the ceruminous glands are found in the external auditory canal in humans [2, 13]. Ceruminous gland neoplasms in the middle ear cavity are extremely rare, and it has been suggested that these tumors originate from ectopic ceruminous glands or arise from the mucosal lining of the middle ear [8, 9]. Ceruminous gland neoplasms can be categorized into benign tumors, which consist of ceruminous adenoma, ceruminous pleomorphic adenoma, and ceruminous syringocystadenoma papilliferum, and malignant tumors, which consist of ceruminous adenocarcinoma, ceruminous adenoid cystic carcinoma, and ceruminous mucoepidermoid carcinoma [2]. Among these, ceruminous gland adenoma is histologically characterized by cuboid and cylindrical cells with an eosinophilic cytoplasm and hyperchromatic round nuclei, without mitotic figures [2]. Macroscopically, the tumor is fibrotic, gray or pink colored, and poorly vascularized. Although ceruminous gland adenomas in the middle ear cavity are usually confined in the middle ear without erosion of the ossicles or bony labyrinth [10], the mastoid cavity can also be involved [4]. Involvement of the Eustachian tube, as observed in our patient, may occur infrequently [4].

Progressive aural fullness, hearing loss, otalgia, and otorrhea are known as typical symptoms of middle ear neoplasm [4–10]. Our patient received medical treatment including oral antibiotics for several months under the diagnosis of otitis media and underwent ventilation tube insertion before visiting our clinic. However, despite prolonged treatment, hearing loss was progressively aggravated. The large air-bone gap, which was observed in the affected ear of our patient, may be attributed to middle ear effusion and attachment of tumor to the malleus as observed in Figure 1. Pure tone audiometry showed mild high-frequency sensorineural hearing loss on both ears, which may be explained as age-related hearing loss. In our patient, tympanomastoidectomy was performed after TBCT evaluation, and ceruminous gland adenoma was histologically diagnosed.

## 4. Conclusions

The present study demonstrated that ceruminous gland adenoma, which originated from the middle ear, can obstruct the bony portion of the Eustachian tube and cause progressive conductive hearing loss and otomastoiditis. Thus, though extremely rare, the middle ear ceruminous gland adenoma should be taken into consideration in the differential diagnosis when conductive hearing loss and otitis media are progressively worsening and do not respond to long-term conventional treatment.
